# Anchoring in Numeric Judgments of Visual Stimuli

**DOI:** 10.3389/fpsyg.2016.00225

**Published:** 2016-02-23

**Authors:** Linda Langeborg, Mårten Eriksson

**Affiliations:** Faculty of Health and Occupational Studies, Department of Social Work and Psychology, University of GävleGävle, Sweden

**Keywords:** anchoring effects, decision making, age estimation, cognitive load, judgment, source credibility

## Abstract

This article investigates effects of anchoring in age estimation and estimation of quantities, two tasks which to different extents are based on visual stimuli. The results are compared to anchoring in answers to classic general knowledge questions that rely on semantic knowledge. Cognitive load was manipulated to explore possible differences between domains. Effects of source credibility, manipulated by differing instructions regarding the selection of anchor values (no information regarding anchor selection, information that the anchors are randomly generated or information that the anchors are answers from an expert) on anchoring were also investigated. Effects of anchoring were large for all types of judgments but were not affected by cognitive load or by source credibility in either one of the researched domains. A main effect of cognitive load on quantity estimations and main effects of source credibility in the two visually based domains indicate that the manipulations were efficient. Implications for theoretical explanations of anchoring are discussed. In particular, because anchoring did not interact with cognitive load, the results imply that the process behind anchoring in visual tasks is predominantly automatic and unconscious.

## Introduction

Estimations of unknown quantities in our environment are a part of everyday life. For example, estimating the age of a new acquaintance or estimating how many cookies there are left in the cookie jar are activities that most people have performed. Such numerical estimations are often influenced by available anchors or standards. If someone was told another person’s estimation of the age of a new acquaintance or of how many cookies there are left in the jar, their estimate would probably be influenced by the other person’s estimates.

Anchoring effects – the assimilation of numeric estimates to previously considered standards (i.e., anchors) – are a robust finding from studies of heuristics and biases in judgments under uncertainty (as in the classical paradigm initiated by Tversky and Kahneman, 1974). The effects occur although participants are told to disregard from the anchor value in their estimates (Mussweiler and Strack, 1999), when anchors are presented without explicit instructions to compare the anchor value with the target (Brewer and Chapman, 2002), and even when the anchors are presented subliminally (Mussweiler and Englich, 2005).

Several psychological mechanisms have been suggested to explain anchoring. Tversky and Kahneman (1974) originally treated anchoring as a judgmental heuristic and proposed that anchoring is the result of *insufficient adjustment* from the anchor. However, studies (e.g., Schkade and Johnson, 1989; Chapman and Johnson, 2002) have failed to find evidence of adjustment in the standard anchoring paradigm. Epley and Gilovich (2001, 2004, 2005, 2006) have questioned insufficient adjustment as a general explanation for the anchoring effect, by showing that adjustment only seems to occur in tasks requiring participants to base their judgment on anchors that are self-generated, rather than provided by the experimenter. Whereas experimenter-provided anchors are values that participants are told explicitly to compare their estimate with (e.g., “Is the population of Chicago more or less than 200,000?”), self-generated anchors are values used spontaneously by the participant as starting points from which they adjust their estimate, without instructions from the experimenter (e.g., for a question about the freezing point of vodka, participants may anchor their estimate in the freezing point of water which they, supposedly, are familiar with). Adjustment is seen as effortful and cognitively demanding, and is thereby affected (i.e., by being terminated too early) by factors that diminishes the ability to dedicate cognitive resources to a task. Epley and Gilovich (2006) manipulated cognitive load, motivation, and even moderate intoxication and showed that these factors affected adjustment from self-generated anchors, although they did not influence responses in the standard anchoring paradigm. The effect of cognitive load on anchoring to self-generated anchors was interpreted as evidence of the same cognitive resources being taxed.

In the standard anchoring paradigm, with experimenter-provided anchors, anchors have been suggested to increase the availability of anchor-consistent information by causing people to recruit biased pools of information (Chapman and Johnson, 1994, 1999; Strack and Mussweiler, 1997). The *Selective Accessibility Model* by Strack and Mussweiler (1997), Mussweiler and Strack (1999) posits that anchoring is produced by enhanced accessibility of information consistent with the suggested anchor. Respondents in the standard paradigm are typically asked to answer a comparative question (i.e., “Is the target value more or less than the anchor value?”) and then to give an absolute estimate of the target value. The model suggests that participants in the standard paradigm test the hypothesis that the target value is equal to the anchor value and that by testing this hypothesis, participants will selectively search for information indicating that the target is similar to the anchor, which enhances the accessibility of information consistent with the anchor value. Cognitive processes involving hypothesis testing are usually considered as cognitively demanding (Mussweiler and Strack, 2001) and would therefore be sensitive to cognitive load, although the activation of semantic knowledge related to the target has been compared with semantic priming (Mussweiler and Strack, 1999) and thus may be regarded as a rather automatic process.

Judgments have also been shown to be affected by anchors semantically unrelated to the absolute question (Chapman and Bornstein, 1996; Wilson et al., 1996; Wong and Kwong, 2000; Oppenheimer et al., 2008). Because semantic activation is not involved in these situations, the applicability of the Selective Accessibility Model in such situations has been questioned. Instead, simple *numeric priming* has been suggested to produce anchoring in these situations, and several researchers propose explanations of anchoring that focus on the anchors’ numerical values or magnitude rather than their semantic meaning (e.g., Wong and Kwong, 2000; Oppenheimer et al., 2008; Frederick and Mochon, 2012; Sleeth-Keppler, 2013). However, as numeric priming seems to be unable to account for findings showing that anchoring is dependent on changes in the judgmental dimension of the target (e.g., smaller effects of anchor values indicating the weight rather than the height of the Brandenburg Gate, when the height is to be estimated, Strack and Mussweiler, 1997), several researchers (e.g., Mussweiler and Strack, 2001; Oppenheimer et al., 2008) propose that different kinds of priming are activated in different situations, or in some cases even simultaneously.

Anchoring has been predominantly researched in answers to general knowledge questions (e.g., Tversky and Kahneman, 1974; Mussweiler and Strack, 1999; Epley and Gilovich, 2001), but anchoring effects have been demonstrated in a number of other areas as well, including probability estimations (Chapman and Johnson, 1999), legal judgments (Englich and Mussweiler, 2001), and forecasting (Critcher and Gilovich, 2008) – see Furnham and Boo (2011) for a review. All these domains are rather abstract and rely on semantic knowledge. Even though tasks such as age estimation or estimation of how many cookies that remain in the jar certainly involve estimation of uncertain numerical quantities, the standard anchoring paradigm has not yet been applied to such simple judgments (but see Sörqvist et al., 2011 for research on the own-anchor effect). The present study extends previous research by exploring anchor effects in judgments that are supported by concrete visual stimuli from pictures, namely age estimates of men and estimation of quantities of items in a container.

Age estimations are particularly interesting in the anchoring domain for several reasons. First of all, the estimates are generally supported by visual stimuli. Moreover, age estimates are regularly exercised in our daily lives, and human age is also restricted to a rather narrow span (few people are over 100 years old). Consequently, age estimates are generally quite accurate (see Rhodes, 2009, for a review), and are thus associated with higher certainty than estimates to the tasks typically used in the standard anchoring paradigm. Of course, age estimates are rarely 100% correct, and some uncertainty always remains, but while uncertainty in the value to be estimated is a classical assumption of anchoring, it is presently unclear whether anchor effects will prevail when judgments become more accurate. We therefore include age estimates in the present study to determine whether standard anchoring effects extend to judgments made under higher certainty than in the classical paradigm. Possible effects of anchoring in age estimation are also of applied importance, as eyewitnesses often are asked to estimate the age of the perpetrator (van Koppen and Lochun, 1997; Pozzulo and Warren, 2003).

Estimations of quantities of objects are, like age estimations, supported by visual stimuli. However, estimations of quantities of objects are not restricted to a specific range of values and there is no reason to assume they are made with higher certainty than other tasks typically employed in the anchoring literature. Furthermore, while age estimates also rest on some semantic knowledge (e.g., of the normal aging process), estimation of quantities of items in a container lacks semantic content. This makes quantity estimations crucial for the Selective Accessibility Model. If anchoring occurs although there are no anchor-consistent cues to be made accessible, anchoring in this domain cannot possibly be attributable to the Selective Accessibility Model, which would reduce the generalizability of the model. Instead, such results would be indicative of perspectives focused more on priming of numbers or magnitudes (e.g., Wilson et al., 1996; Wong and Kwong, 2000; Oppenheimer et al., 2008). Also, the unique characteristics of estimation of quantities (the visual dependence and low semantic content) means that the finding of anchor effects in quantity estimations would further extend the previously assumed boundaries of anchoring.

In the present study, cognitive load is manipulated to explore the anchoring processes in the two new visual domains and to compare the effects to that of anchoring in responses to general knowledge questions. Differences in the effects of cognitive load between domains would be indicative of different processes. Cognitive load serves here as an indication of whether anchoring is driven by cognitively demanding processes such as successive adjustment, or by more automatic and unconscious processes. Also, although Epley and Gilovich (2006) have shown that cognitive load does not affect anchoring to experimenter-provided anchors, they measured anchoring in terms of adjustment (by subtracting the estimation from the anchor value), rather than in more conventional anchoring measures. In the present study, we investigate if cognitive load interact with anchoring to experimenter-provided anchors, measured more conventionally, by mean differences between estimates based on high anchors and estimates based on low anchors. Because cognitive processes involving hypothesis testing are considered as cognitively demanding (Mussweiler and Strack, 2001), an effect of cognitive load would be expected for such tasks. However, as the activation of semantic knowledge related to the target has been compared to semantic priming (Mussweiler and Strack, 1999), it may be regarded as a rather automatic process and no effect of cognitive load would thus be expected. The effect of cognitive load on judgments within the selective accessibility paradigm is therefore still an open question.

In addition, the effect of source credibility is investigated. In experiments on anchoring, participants are generally informed that the anchors are randomly selected. The argument for this is that, without this information, the participants would assume that the experimenter has good reasons for presenting that particular value and that it might serve as a clue to the right answer (in adherence to the maxim of communication relevance that posits that not only is all relevant information given in a conversation but also that all given information is relevant, Grice, 1975). Although it is well known that anchoring occurs even with this information about randomly selected anchors, no study has yet investigated whether or not information about randomly selected anchor values actually is associated with weaker anchoring, compared to no information about anchor selection. Recent work (Wegener et al., 2010; Dowd et al., 2014) indicates that participants do take information about anchor origin into account, because participants show higher confidence and place higher weight on the anchor in answers to general knowledge questions when it comes from high-credible (estimators knowledgeable in the domain) compared with low-credible (estimators not knowledgeable in the domain) sources. Glöckner and Englich (2015) have also found effects of relevance in anchoring in juridical decisions, with stronger anchor effects for relevant (concerning the case which sentence were to be decided) than irrelevant (concerning a completely different case) anchors.

To investigate the effect of information about source credibility on anchoring, participants in the present study were given different information as to the selection of the anchor values: one group was given information that the anchor values were randomly chosen (low credibility group); one group was given information that the anchor values were answers from an expert in the relevant domain (high credibility group); and one group was not given any information about the selection of the anchor values (control group). Based on earlier studies (Wegener et al., 2010; Dowd et al., 2014), an interaction between source credibility and anchor effects would be expected, with larger anchoring effects for participants given information that the anchor values were answers from an expert in the field than for participants given information that the anchor values were randomly chosen. However, because the two new domains in our study are concrete and visually based, and associated with lower degrees of uncertainty than in the abstract tasks used earlier, it can be argued that the participants have a better chance to make their own judgments in these domains. This opens for participants to question the expert’s expertise and put less weight to their answers (i.e., the anchor values). Thus, it is not clear what effect the introduction of anchor values provided by an expert will have on the two visual domains tasks. For the abstract general knowledge tasks, however, an interaction between source credibility and anchoring, as described above, was expected.

In sum, the purpose of the present study was to investigate anchor effects in two visual domains with different levels of certainty and different levels of semantic content. Cognitive load was manipulated to explore possible differences in the processes behind anchoring in the different domains. If the process behind anchoring in the two visual domains is similar to the process behind anchoring in answers to general knowledge questions, the effect of cognitive load should be the same for each of the researched domains. Also, we investigated the effect of the often used information about random selection of anchor values by manipulating source credibility. Higher effects of anchoring were hypothesized when the anchor value came from an expert compared to randomly generated values, at least for the general knowledge tasks.

## Materials and Methods

### Participants

Totally, 144 students at the University of Gävle (90 women and 54 men) with a mean age of 24 years (range = 18–49 years) served as participants and received a cinema voucher for participation. All participants were healthy adults and participated under informed consent (confirmed by signing a form). The experiment caused no harm to any part and the identity of the participants has been kept confidential. All ethical guidelines given by the American Psychological Association were respected.

### Materials

Estimations in the general knowledge domain were based on eight knowledge questions used by Jacowitz and Kahneman (1995), but adapted to a Swedish context. Age estimations were based on eight full body photographs of men aged 28–47 years. Quantity estimations were based on photographs of eight glass containers with different items (e.g., beads and nails). See **Table 1** for details of the material.

**Table 1:** Questions and stimuli used in the study.

	Actual value	Low anchor	High anchor
**General knowledge questions**			
Length of Göta Kanal (a Swedish canal)	191 km	134	248
Population of Växjö (a Swedish town)	85 000	59 500	110 500
Height of Kilimanjaro	5 895 m	4 127	7 664
Number of babies born in Sweden in 2013	112 286	78 600	145 972
Height of tallest oak tree in Sweden	36 m	25	47
Meat eaten/year by the average Swede	49 kg	34	64
Distance from Luleå-Falun (Swedish towns)	830 km	581	1 079
Maximum speed of house cats	50 km/h	35	65
**Items for quantity estimations**			
Macaroni	663	464	862
Cashew nuts	166	116	216
Plus–Plus (building blocks for kids)	151	106	196
Small beads	268	188	348
Carpentry nails	137	96	178
Stones	95	67	124
Maxi beads	378	265	491
Matches	114	80	148
**Items for age estimation**			
35 years old (two males)	35	25	46
47-year-old male	47	33	61
28-year-old male	28	20	36
46 years old (two males)	46	32	60
33-year-old male	33	23	43
42-year-old male	42	29	55

### Design and Procedure

The experiment had a 2 (Anchor value: high or low) × 2 (Cognitive load: load or no load) × 3 (Source credibility: high, low, or control) repeated measure design with Anchor value and Cognitive load as within-subject variables and Source credibility as between-subject variable. The experimental design was the same for all three judgmental domains (age estimation, quantity estimation, and general knowledge questions).

The participants sat alone in front of a laptop in a silent laboratory. The experimental instructions (which included information that the participants for each item were to decide whether the target value was more or less than a comparison value) were identical for all participants, but additional information differed depending on the experimental condition (Source credibility). The low credibility group was informed that the comparison values were randomly selected and therefore not informative of the actual values of the targets (following a commonly used method to reduce ascribed informativeness of the anchors, e.g., Strack and Mussweiler, 1997; Wegener et al., 2001). The high credibility group was given the following information: “The comparison values are answers from an expert on this type of judgments. The expert’s answers have previously been shown to be close to the actual values of the targets in similar tests. However, that does not necessarily mean that the expert’s answers are as accurate in this test.” The control group received no information about the choice of anchor values.

Next, participants were asked to complete three tasks (estimations of age, estimations of quantities, and answers to general knowledge questions) in a block design. The order of the blocks was counterbalanced across participants.

For each item, participants were first asked to answer a comparative question about whether the target value was higher or lower than a comparison value (anchor value) and were then asked to provide an absolute estimate of the target value. A low anchor was used in half of the trials and a high anchor was used in the other half. Anchor values (high and low) for each item were set at 130% and 70% of the true target value, respectively, and were counterbalanced across blocks and participants.

Cognitive load was manipulated by having participants memorize a letter string presented before each question (see Kruger, 1999; Gilbert, 2002; Epley and Gilovich, 2006) for half of the estimates. Before the comparative question, participants were informed that on the upcoming screen they would be presented with either a blank screen or a string of eight consonants that they should memorize and rehearse. The importance of trying to remember the letters as well as possible was emphasized. The blank screens/letter strings were presented for 7 s. Next, participants were first given the comparison question and the absolute question and then asked to report the letters (if any) they were given earlier. This cycle was repeated for each item, with half of the items of each estimation type (age estimation, quantity estimation, or general knowledge questions) being presented under cognitive load and half without. The order of the conditions (cognitive load/no cognitive load) was counterbalanced across participants.

### Dependent Variables and Statistics

Unreasonable estimations (e.g., an estimation of the number of babies born in Sweden in 2013 as 98 when the correct value is 112 286) were classified as keyboard slips and removed before the analysis. Together with missing data, 2.2% of the data were lost, and replaced with the mean value for the corresponding condition (i.e., a missing estimate from a female participant estimating age under cognitive load in the high credibility condition was replaced with the mean value for all female participants’ age estimations under cognitive load in the high credibility condition). After that, 1.2% of the data were identified as outliers by using the outlier labeling rule with g’ set at 2.2 (Hoaglin and Iglewicz, 1987). These data were replaced with the mean value for the right condition (see above). Because different items in each domain (particularly in general knowledge questions) were estimated on different scales, quota values (estimated values divided by true values) were used as the dependent variable in the analyses. Quota values under 1 represent underestimations, quota values over 1 represent overestimations and quota values equal to 1 represent accurate estimations.

## Results

### General Knowledge

Participants gave generally higher estimates to the general knowledge questions when given high anchors (*M* = 1.17, *SD* = 0.25) than when given low anchors (*M* = 0.74, *SD* = 0.15). This was confirmed by a 2 (Anchor value: high or low) × 2 (Cognitive load or no load) × 3 (Source credibility: high, low, or control) Analysis of variance with Anchor value and Cognitive load manipulated within subjects and Source credibility manipulated between subjects. The analysis revealed a significant main effect of Anchor value [*F*(1,141) = 318.5, *p* < 0.001, $\eta_{p}^{2}$ = 0.69], but no interactions between Source credibility and Anchor value or between Cognitive load and Anchor value. The main effects of Cognitive load and Source credibility did not reach statistical significance.

### Age Estimation

For age estimates, participants generally produced higher age estimates when given high anchors (*M* = 1.10, *SD* = 0.10) than when given low anchors (*M* = 0.93, *SD* = 0.11), *F*(1,141) = 162.52, *p* < 0.001, $\eta_{p}^{2}$ = 0.54. There was also a main effect of Source credibility [*F*(2,141) = 6.26, *p* = 0.002, $\eta_{p}^{2}$ = 0.08], such that participants in the high credibility group gave higher estimates (*M* = 1.04, *SD* = 0.07) than participants in both the low credibility (*M* = 1.00, *SD* = 0.06) and control group (*M* = 1.00, *SD* = 0.06). The failure to find an interaction between Anchor value and Source credibility indicates that the magnitude of the anchoring effect did not vary with different levels of Source credibility. There was no effect of Cognitive load, nor any interaction between Anchor value and Cognitive load. Thus, age judgments are no different from general knowledge questions in this respect, despite the difference in task characteristics.

### Quantity Estimations

Participants gave generally higher quantity estimates when given high anchors (*M* = 0.80, *SD* = 0.27) than when given low anchors (*M* = 0.60, *SD* = 0.14), *F*(1,141) = 112.79, *p* < 0.001, $\eta_{p}^{2}$ = 0.44. There was also an effect of Source credibility [*F*(2,141) = 3.99, *p* = 0.021, $\eta_{p}^{2}$ = 0.05], revealing that participants in the high credibility condition gave higher estimates (*M* = 0.76, *SD* = 0.19) than participants in the control group (*M* = 0.65, *SD* = 0.18). A difference in anchoring magnitude depending on Source credibility was not supported in that there was no interaction between Anchor value and Source credibility. A main effect of Cognitive load was also found in these estimations [*F*(1,141) = 10.45, *p* = 0.002, $\eta_{p}^{2}$ = 0.07]. More specifically, participants made lower estimates under cognitive load (*M* = 0.68, *SD* = 0.21) than without (*M* = 0.73, *SD* = 0.20). A difference in anchoring magnitude depending on Cognitive load was not supported as indicated by the absence of an interaction between Anchor value and Cognitive load. Hence, anchoring in quantity estimations behave in similar ways as anchoring in age estimations and responses to general knowledge questions.

## Discussion

Strong effects of anchoring were found in all three judgmental domains under study, irrespective of cognitive load and information about source credibility. Thus, we show that anchoring can occur even in domains that are strongly visually based and for estimations made with rather high certainty (age estimations) as well as for estimations with a low level of semantic content (quantity estimations). Our results are compatible with the process behind anchoring being predominantly automatic and unconscious. The fact that the present study manipulated anchor values within subjects instead of between subjects as in most other studies on anchoring makes the contribution particularly strong. In addition, the design of employing repeated measures in each domain indicates high reliability of the findings.

The lack of an interaction between cognitive load and anchoring on general knowledge questions corroborates earlier research by Epley and Gilovich (2006), which has shown that cognitive load does not affect anchoring (measured in terms of adjustment) to experimenter-provided anchors. Here, we extend this research with a more traditional measure of anchoring. The results for anchoring in all three domains under investigation are compatible with perspectives that regard anchoring as originating from System 1 processes, processes that are fast, automatic and unconscious (e.g., Chapman and Johnson, 1999; Frederick et al., 2010).

The phenomenon of subjects being influenced by anchors in their answers to general knowledge questions even when the anchors are said to be randomly generated is well-documented (e.g., Tversky and Kahneman, 1974; Strack and Mussweiler, 1997; Chaxel, 2014). Nevertheless, whether there is a difference in the magnitude of the anchor effect between standard information about randomly chosen anchor values and no information at all has never been investigated before. We found no indication of such a difference as there was no interaction between source credibility and anchoring in any of the judgmental domains. Thus, based on our findings, the practice of informing the participants that the anchors are randomly selected seems ineffective. Although the lack of interaction between source credibility and anchoring seems somewhat reasonable in regards to estimations in the two visual domains, where the subjects have the object to be estimated right in front of their eyes, it is noteworthy that it for answers to general knowledge questions did not matter even whether the anchors in the present study were said to be randomly generated or originated from estimates by an expert in the field. This is in contrast to Dowd et al. (2014) who found that participants placed higher weight on the anchor in their answer to a question about distances between cities when the anchor was said to come from geography students compared with fine arts students. However, Dowd et al. (2014) analyzed their data using a “weighting average” and not in terms of classical anchor effects. Also, their questions were supposedly more difficult than the ones used in our study, which may have made their participants more willing to accept the suggestions from the high-credible sources. Wegener et al. (2010) discussed another study in which participants showed more anchoring to credible than to non-credible sources, but because they referred to unpublished raw data, no conclusions on possible explanations to the difference in results can be drawn. At this point, data on the effect of source credibility on anchoring are inconclusive. However, it is an important issue because an interaction of source credibility and anchoring would be hard to conceive as being the result of an automatic and unconscious process. Therefore, more research is needed on the effects of source credibility on anchoring.

The credibility of the expert can be instantiated in more detail than what was done in our study, with details that might increase the trustworthiness of her expertise. For example, one can elaborate more on the nature of her expertise by describing her previous experience (c.f. Glöckner and Englich, 2015). There is also a chance that an anchor point closer to the targets’ true values would appear as more credible, at least for age estimates. But as there was no interaction with credibility for any domain, it is not likely that the distance between the true value and the anchor provided by the expert was responsible for the lack of interaction with source credibility in this study.

This is the first demonstration of classical anchor effects in age estimation. It is of theoretical importance because age estimates, in contrast to previously researched domains, are strongly visually based and made with rather high certainty. Thus, the boundary conditions for anchoring have been further extended. The absence of an interaction between cognitive load and anchoring in age estimations suggest that the anchoring processes in this domain is similar to the process in classical anchoring tasks (e.g., general knowledge questions). Effects of anchoring in age estimation are also of applied importance. Eyewitnesses are often asked to estimate the age of the perpetrator (van Koppen and Lochun, 1997; Pozzulo and Warren, 2003), and if the witness hesitates in their estimation, the police may ask further questions including suggestions or comparative questions which may lead the witness to anchor their estimation to the proposed value. Because of this, effects of anchoring should be considered when questioning eyewitnesses of information such as the age of a culprit.

Evidence of anchoring in quantity estimations is important, as quantity estimations are mainly based on visual stimuli and are low in semantic cues, which together with the results on age estimations extend the boundary conditions for anchoring. Importantly, because there was little semantic information at hand in the quantity estimations, anchoring in quantity estimations can hardly be explained by the Selective Accessibility Model. Instead, more numerically focused perspectives (e.g., Wong and Kwong, 2000; Oppenheimer et al., 2008; Frederick and Mochon, 2012; Sleeth-Keppler, 2013) seem better fitted to explain anchoring in these judgments. Previous results questioning the Selective Accessibility Model have mainly used methods other than the ones used in the standard anchoring paradigm. Our results add to previous findings by demonstrating situations where the Selective Accessibility Model is not applicable, despite the use of the standard anchoring procedure.

## Author Contributions

LL and ME designed the experiment. LL performed the data collection and the data analysis. LL and ME wrote the manuscript.

## Conflict of Interest Statement

The authors declare that the research was conducted in the absence of any commercial or financial relationships that could be construed as a potential conflict of interest.
